# Emerging roles of Toll-like receptor 9 in cardiometabolic disorders

**DOI:** 10.1186/s41232-020-00118-7

**Published:** 2020-07-21

**Authors:** Sachiko Nishimoto, Daiju Fukuda, Masataka Sata

**Affiliations:** 1 Department of Cardiovascular Medicine, Tokushima University Graduate School of Biomedical Sciences, 3-18-15, Kuramoto-cho, Tokushima, 770-8503 Japan; 2 Department of Cardio-Diabetes Medicine, Tokushima University Graduate School of Biomedical Sciences, Tokushima, 770-8503 Japan

**Keywords:** Toll-like receptor 9, Cell-free DNA, Inflammation, Cardiometabolic disorders

## Abstract

Growing evidence suggests that damage-associated molecule patterns (DAMPs) and their receptors, pattern recognition receptors (PRRs), are associated with the progression of cardiometabolic disorders, including obesity-related insulin resistance and atherosclerosis. Cardiometabolic disorders share sterile chronic inflammation as a major cause; however, the exact mechanisms are still obscure. Toll-like receptor 9 (TLR9), one of the nucleic acid-sensing TLRs, recognizes DNA fragments derived from pathogens and contributes to self-defense by activation of the innate immune system. In addition, previous studies demonstrated that TLR9 recognizes DNA fragments released from host cells, accelerating sterile inflammation, which is associated with inflammatory diseases such as autoimmune diseases. In obese adipose tissue and atherosclerotic vascular tissue, various stresses release DNA fragments and/or nuclear proteins as DAMPs from degenerated adipocytes and vascular cells. Recent studies indicated that the activation of TLR9 in immune cells including macrophages and dendritic cells by recognition of these DAMPs promotes inflammation in these tissues, which causes cardiometabolic disorders. This review discusses recent advances in understanding the role of sterile inflammation associated with TLR9 and its endogenous ligands in cardiometabolic disorders. New insights into innate immunity may provide better understanding of cardiometabolic disorders and new therapeutic options for these major health threats in recent decades.

## Introduction

**Fig. 1:**
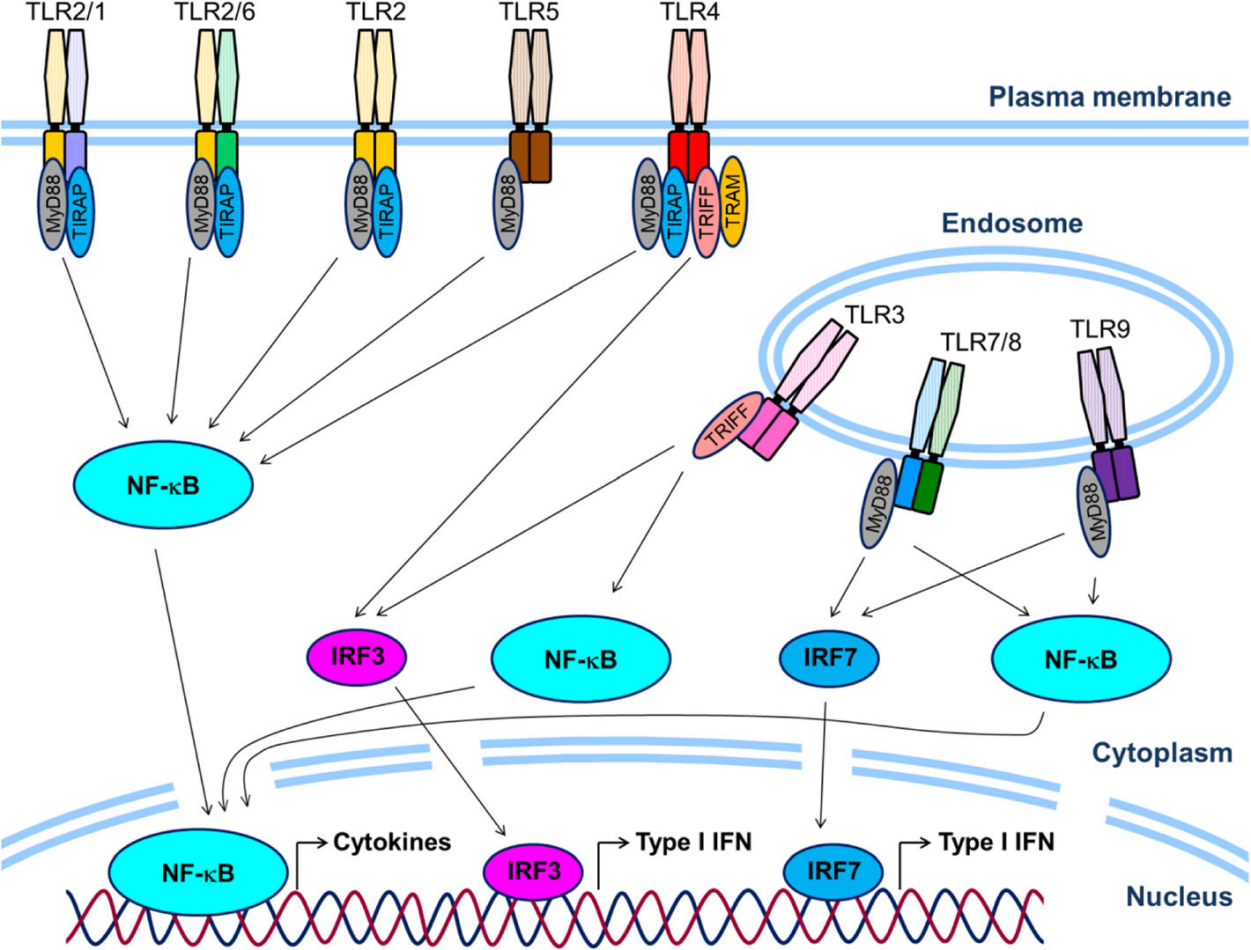
Overview of TLR signaling. TLRs are classified into cell surface TLRs and intracellular TLRs. Each TLR recognizes their specific ligands and promotes gene expression of inflammatory molecules mainly via NF-κB and IRF pathway in immune cells

Immune cells such as macrophages recognize the structures of pathogens through a family of pattern recognition receptors (PRRs) such as Toll-like receptors (TLRs) by detecting components referred to as pathogen-associated molecular patterns (PAMPs) and activate the innate immune system for self-defense [1]. TLRs are evolutionarily conserved proteins. Thus far, 10 functional TLRs have been identified in humans and 12 in mice. TLRs are classically categorized into two groups by their localization in/on the cell: cell surface TLRs include TLR1, TLR2, TLR4, TLR5, TLR6, and TLR11, which mainly recognize membrane components such as lipids, lipoproteins, and proteins on bacteria; intracellular TLRs include TLR3, TLR7, TLR8, and TLR9, which recognize viral and bacterial nucleic acids. The latter group is expressed in intracellular vesicles such as endosomes, lysosomes, and endoplasmic reticulum (ER) [2]. Numerous previous studies have examined the mechanisms underlying TLR signaling and demonstrated that it requires the recruitment of several adaptor molecules, leading to the activation of the NF-κB and interferon (IFN) regulatory factor (IRF) pathways, which accelerate inflammatory responses (Fig. 1). In addition, emerging evidence has revealed that TLR signaling is involved in not only innate immune systems but also in the pathogenesis of various diseases such as autoimmune diseases and lifestyle-associated diseases. Among them, the role of TLRs in the pathogenesis of cardiometabolic disorders, one of the health threats for humans in recent decades, has attracted much attention. TLRs whose roles in cardiometabolic disorders have been most studied are TLR2 and TLR4 [3–9], while recent studies have suggested the participation of TLR9, originally known as a sensor for exogenous DNA fragments, in these diseases (Table 1). This review briefly summarizes the role of TLR9 in the pathogenesis of inflammatory diseases and describes recent findings, including our own, on the potential participation of TLR9 in the development of cardiometabolic disorders.

**Table 1:** Potential ligands and roles of TLR9 in cardiometabolic disorders

Organ	Ligand	Role of TLR9 in cardiometabolic organs	Models used	Ref
Adipose tissue	DNA fragments	- Induction of insulin resistance - Induction of adipose tissue inflammation	- HFD feeding - WT and *Tlr9*^-/-^ mice - TLR9 antagonist - Murine peritoneal macrophages - Human study	[10]
	Nucleic acids	- Induction of insulin resistance - Induce adipose tissue and liver inflammation	- HFD feeding - WT mice - Inhibitors of ET formation or a TLR7/9 antagonist	[11]
		- Up-regulation of inflammatory cytokines and chemokines	- HFD feeding - *ob/ob* and WT mice	[12]
		- Improvement of insulin resistance* - Reduction of adipose tissue inflammation*	- HFD feeding - WT and *Tlr9*^-/-^ mice	[13]
Liver	HMGB1	- Increase of body weight gain - Increase of hepatic inflammation	- HFD feeding - WT and *Tlr9*^-/-^ mice - Anti-HMGB1 antibody	[14]
	mtDNA	- Increase of NAFLD activity - Induction of liver inflammation	- HFD feeding - WT, *Tlr9*^-/-^, and macrophage-specific Tlr9-/- mice - TLR9 antagonist - Human study	[15]
		- Increase of non-apoptotic hepatocyte death - Promotion of liver fibrosis - Induction of liver inflammation	- HFD feeding - Hepatocyte-specific *DNase 2a*^*-/-*^ mice - TLR9 agonist/antagonist - Murine hepatocyte cell line	[16]
	Not identified	- Stimulation of steatosis, inflammation, and fibrosis - Induction of insulin resistance	- CDAA diet-feeding - WT, *Tlr9*^-/-^, *Il1r*^-/-^, and *Myd88*^-/-^ mice - Murine Kupffer cells	[17]
Vasculature	DNA fragments	- Association with coronary artery disease severity	- Human study	[18]
		- Promotion of atherosclerotic lesion development	- *Apoe*^*-/-*^ and *Tlr9*^-/-^*Apoe*^*-/-*^ mice - Angiotensin II infusion - Murine peritoneal macrophages - Human study	[19]
		- Promotion of atherosclerotic lesion development - Promotion of inflammatory activation of Endothelial cells - Promotion of inflammatory activation of T cells and pDCs	- *Apoe*^*-/-*^ mice - TLR9 agonist - Peripheral blood mononuclear cells - Human study	[20]
	HMGB1	- Promotion of vascular injury-induced neointima hyperplasia - Increase of foam cell accumulation - Promotion of inflammatory activation of macrophages	- WT and *Tlr9*^-/-^ mice - Vascular injury-induced neointima hyperplasia - HMGB-1 and anti-HMGB1 antibody - Murine peritoneal macrophages and RAW264.7 cells	[21]
		- Promotion of vascular injury-induced neointima hyperplasia - Increase of foam cell accumulation - Promotion of inflammatory activation of macrophages	- Apolipoprotein E*3-Leiden mice - Vascular injury-induced neointima hyperplasia - TLR7/9 dual antagonist - Murine BMDMs	[22]
	Not identified	- Promotion of inflammatory activation of macrophages - Promotion of foam cell formation	- Murine peritoneal macrophages and RAW264.7 cells - TLR9 agonist	[23, 24]
		- Promotion of inflammatory activation of pDCs - Induction of plaque destabilization	- Leukocytes collected from human atherosclerotic lesions (pDCs and T cells) - Peripheral blood mononuclear cells - TLR9 agonist	[25]
		- Promotion of atherosclerotic lesion development - Stimulating endothelial dysfunction - Promotion of inflammatory cell accumulation	- *Apoe*^*-/-*^ mice and WT mice - Electric denudation of carotid artery - TLR9 agonist	[26]
		- Inhibition of atherosclerosis development* - Reduction of vascular inflammation* - Reduction of T cell accumulation* - Increase of cholesterol level	- *Apoe*^*-/-*^ and *Tlr9*^-/-^*Apoe*^*-/-*^ mice - TLR9 agonist	[27]
Heart	mtDNA	- Related with the development of heart failure after TAC - Worsen survival after TAC	- Cardiomyocyte-specific *DNase2a*^*-/-*^ mice - TAC - TLR9 antagonist - Adult murine cardiomyocytes	[28]
		- Induction of cardiomyocyte death	- WT and NF-κB luciferase reporter mice - Primary cardiac cells and cardiac fibroblasts - mtDNA and TLR9 agonist	[29]
		-Induction of inflammatory cell activation	- Human study - THP-1 cells, Raji cells, and HUVECs - mtDNA - TLR9 antagonist	[30]

BMDM bone marrow-derived macrophage, CDAA diet choline-deficient amino acid-defined diet, ET extracellular trap, TAC transverse aortic constriction

*Protective role against disease progression

## DNA sensors in innate immunity

Nucleic acids are indispensable for life; however, exogenous nucleic acids, especially those from exogenous organisms such as bacteria and viruses, strongly induce inflammation. There are several sensors for nucleic acids. Generally, TLR3, TLR7/8, and TLR9 recognize double-stranded (ds) RNA, RNA, and DNA, respectively. Other than these TLRs, nucleic acids are also detected by other groups of molecules, such as retinoic acid-inducible gene I (RIG-I) and melanoma differentiation-associated gene 5 (MDA5) by detecting single-stranded (ss) RNA and dsRNA, respectively [31, 32]. In addition to endosomal DNA-sensing proteins, cytoplasmic DNA sensors, such as the cGAMP synthase-cGAMP-stimulator of interferon genes (STING), have been reported [33].

Among these, TLR9 is one of the most studied sensors for nucleic acids. TLR9 recognizes DNA fragments that contain unmethylated CpG DNA and plays a role in innate immunity [34, 35]. TLR9 localizes in the ER in multiple cell types, including macrophages, B cells, dendritic cells, and plasma cells [34]. After uptake of the ligands by phagocytosis, TLR9 immediately redistributes from the ER to the CpG DNA-containing structures. TLR9 activation leads to the production of type I IFN through myeloid differentiation primary response 88 (MyD88)-IRF7 or of inflammatory cytokines through MyD88-NF-κB, accelerating inflammatory responses [2, 36–38].

## Role of TLR9 in inflammatory diseases

**Fig. 2:**
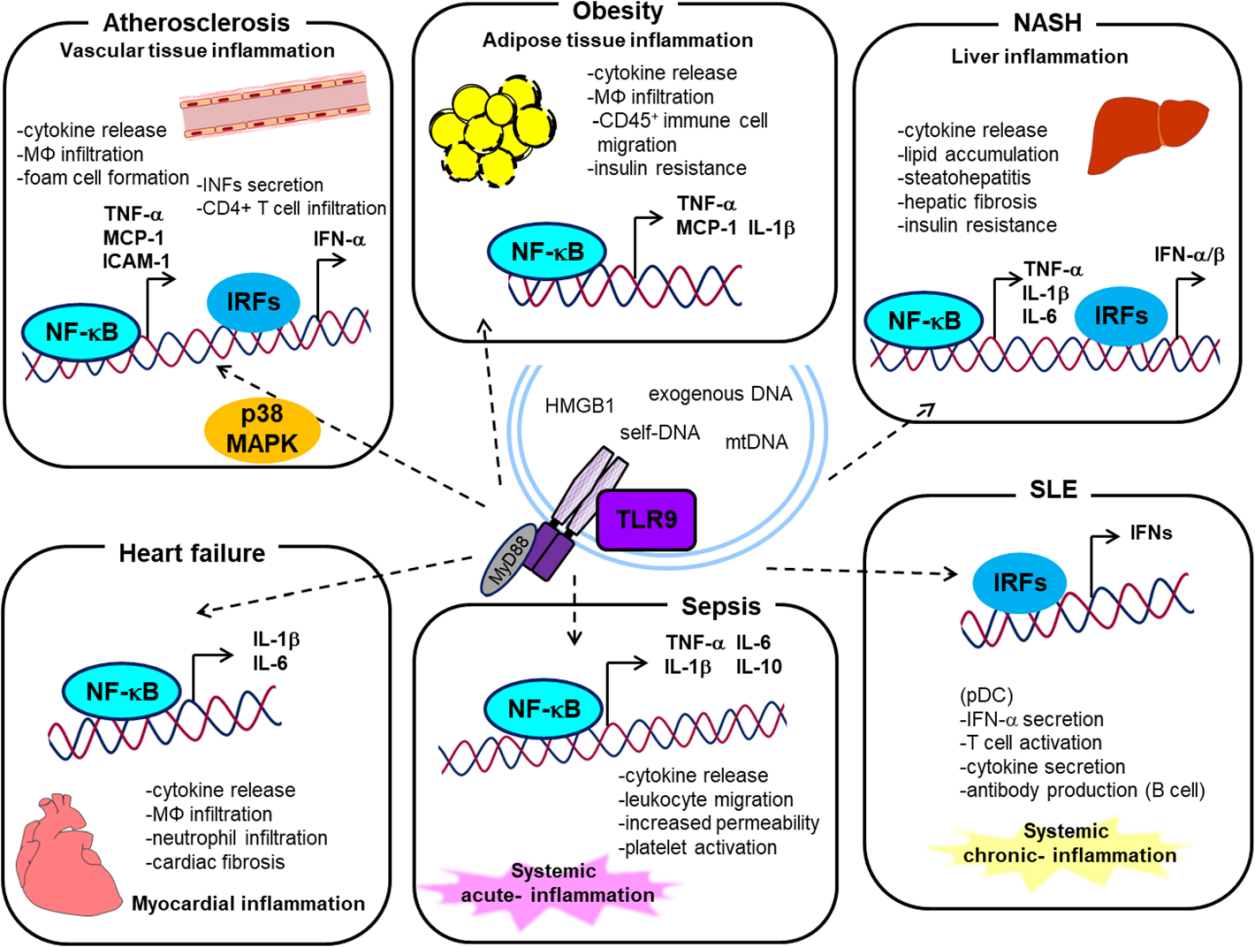
TLR9 signaling in inflammatory diseases. Activation of TLR9 signaling in immune cells leads to release various cytokines and interferons in cardiometabolic organs and other tissues, participating in the pathogenesis of both infectious and sterile inflammatory diseases

TLR9 activation plays a central role in self-defense against exogenous organisms as a sensor for exogenous DNA fragments; however, accumulating evidence has revealed that TLR9 also recognizes self-derived DNA and promotes inflammation improperly in certain disease contexts such as autoimmune diseases [39, 40]. The pathogenesis of autoimmune diseases is not clear. The initiating stimuli are often unidentified, and the reasons why the mechanisms that ordinarily handle the immune response fail are unknown. However, it is clear that these diseases are characterized by an extraordinarily destructive tissue environment. Hence, the DAMP level is elevated locally and/or systemically in these conditions. To avoid unwanted activation of TLR9 by endogenous DNA fragments, its level is thought to be maintained under a certain threshold. However, disease conditions in which an abundant supply of DNA fragments overwhelms the removal mechanisms, or the removal mechanisms of DNA fragments deteriorate even if the supply is maintained, cause elevation of the levels of endogenous DNA fragments. The existence of extracellular DNA in human plasma, also known as cell-free DNA (cfDNA), has been described almost from the 1940s [41], whereas recent studies demonstrated elevation of cfDNA level and its association with the pathophysiology of several inflammatory diseases [42]. For example, circulating cfDNA level is higher in systemic sclerosis [43], experimental pulmonary thromboembolism [44], end-stage renal disease [45], and sepsis [46]. Of note, the role of cfDNA has attracted much attention in the pathogenesis of autoimmune diseases including systemic lupus erythematosus (SLE) and rheumatoid arthritis [47–52]. In fact, autoantibodies against dsDNA and nucleosomes represent a feature of SLE [39, 53, 54]. In general, TLR9 activates IRFs and/or NF-κB, which produces interferons and cytokines, leading to the acceleration of inflammatory responses in these diseases [2, 36–38]. In addition, other studies demonstrated that TLR9 promotes p38 mitogen-activated protein kinase (MAPK) activation and the subsequent NF-κB activation, stimulating inflammation [19, 55]. On the other hand, a few studies reported that ligation of TLR9 with its ligand has beneficial effects on some disease context such as cerebral ischemia/reperfusion injury by activation of PI3K/Akt signaling [56]. These results indicated that the role of TLR9 in inflammatory diseases and the underlying mechanisms are context-dependent and signaling systems under TLR9 might not be fully understood. TLR9-mediated signaling and following response suggested in the inflammatory diseases are summarized in Fig. 2. A number of studies have suggested a link between TLR9 and inflammatory diseases; however, the role of TLR9 in the development of cardiometabolic diseases in which chronic sterile inflammation takes part as an underlying mechanism remains not fully investigated.

## Role of TLR9 in metabolic diseases

Because of the change in our lifestyle, the prevalence of obesity is increasing all over the world. Obesity is closely associated with multiple metabolic abnormalities including insulin resistance, hyperglycemia, dyslipidemia, hepatic steatosis, and hypertension. In the pathobiology of obesity and obesity-related complications, chronic sterile inflammation in metabolic organs plays a central role. The mechanisms by which obesity promotes inflammation in metabolic organs are still unknown, although recent studies suggested the contribution of TLRs [7, 57–59]. For example, adipose tissue is an energy-storing organ, in which interaction of immune cells, hypertrophy and proliferation of adipocytes, and angiogenesis are highly coordinated [60–63]. However, obesity-related conditions, such as higher oxidative stress [62], lower oxygen pressure [63], and enhanced inflammation [60, 64], disturb this balance, leading to the induction of cellular degeneration and enhancement of cellular turnover in adipose tissue [65–67]. A previous study demonstrated that local and/or systemic adipocyte-derived factors contribute to multiple pathological states associated with obesity, including adipose tissue inflammation [68]. Here, TLR2 and TLR4 recognize their ligands from obese and degenerated adipose tissue, mediating adipose tissue inflammation [59, 69–72]. Importantly, clinical studies showed that TLR2/TLR4 expression was increased in adipose tissue and monocytes in obese or diabetic patients, which is correlated with the severity of insulin resistance [5, 73].

**Fig. 3:**
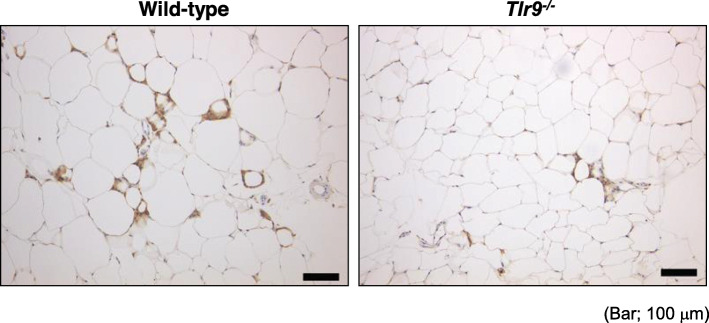
Genetic deletion of TLR9 attenuated obesity-induced adipose tissue inflammation. Representative figures of Mac3 staining of visceral adipose tissue from HFD-fed wild-type or TLR9-deficient mice. Genetic deletion of TLR9 reduced the accumulation of macrophages in adipose tissue, indicating less inflammation. Bar, 100 μm

In addition to other types of endogenous ligands for TLRs, such as saturated fatty acids and heat shock protein (HSP), self-derived DNA fragments are thought to be released. However, the role of self-derived DNA fragments and the contribution of TLR9 to the development of adipose tissue inflammation have not been studied. We previously demonstrated that high-fat feeding increased the level of plasma ssDNA in mice. Also, plasma ssDNA level was higher in patients with visceral obesity diagnosed by computed tomography compared with the non-obese population. We further found that the level of ssDNA positively correlated with HOMA-IR, a parameter of insulin resistance, in humans [10]. The increase in plasma cfDNA in obese subjects was significant but modest compared with previous research on patients with other inflammatory diseases, including cancer and SLE [45, 51, 74]. Importantly, obesity is associated with chronic, low-grade inflammation. This might be one of the explanations for the lower level of cfDNA in obese individuals. Next, we investigated the roles of TLR9 in adipose tissue inflammation. In in vitro studies, a TLR9 agonist, ODN1826, promoted the expression of inflammatory molecules such as tumor necrosis factor-α (TNF-α) and monocyte chemoattractant protein-1 (MCP-1), important molecules for adipose tissue inflammation, in macrophages. Also, cfDNA collected from degenerated adipocytes activated macrophages through TLR9 and stimulated the expression of inflammatory molecules [10]. Obesity induced by high-fat diet (HFD) feeding promoted TLR9 expression in adipose tissue in addition to cfDNA in animal studies [10, 12]. Genetic deletion of TLR9 decreased the accumulation of macrophages in obese adipose tissue (Fig. 3) and inhibited the development of obesity-induced adipose tissue inflammation and insulin resistance. Furthermore, bone marrow-specific expression of TLR9 worsened insulin resistance under HFD feeding compared with that in mice lacking TLR9 in their body. On the other hand, administration of an inhibitory oligonucleotide for TLR9, iODN2088, to HFD-fed wild-type mice attenuated inflammation in adipose tissue and improved insulin resistance. These results suggest a link between TLR9 and obesity-associated insulin resistance, and the potential of cfDNA-TLR9 signaling as a therapeutic target. In addition, in the development of obesity-associated metabolic disorders, several reports suggest the role of another endogenous ligand of TLR9 beside self-derived DNA fragments. In a clinical study of obese individuals, Guzmán-Ruiz et al. showed an elevated level of high mobility group box protein-1 (HMGB1) in the plasma as well as increased expression in visceral adipose tissue, which correlated with markers of adipose tissue inflammation [75].

A role of TLR9 in the pathogenesis of non-alcoholic steatohepatitis (NASH) has also been reported. In mice, a TLR7/9 antagonist (IRS954) was effective for improving hepatic steatosis and NASH [15]. TLR9-deficient mice had less insulin resistance than wild-type mice on a choline-deficient amino acid-defined diet [17]. Liver damage including mitochondrial stress promotes mitochondrial DNA (mtDNA) leakage. Similar to bacterial DNA, mtDNA contains a predominantly unmethylated CpG motif [76, 77] and can act on macrophage TLR9, leading to a strong induction of inflammatory responses. Saito et al. demonstrated mtDNA-mediated activation of the TLR9/IFN-β signal pathway accelerates non-apoptotic hepatocyte death and liver fibrosis [16]. In addition to animal studies, a clinical study demonstrated that the number of mtDNA copies was 3.2-fold higher in NASH patients than in healthy controls [78]. Of note, recent studies have established that mtDNA triggers various inflammatory or degenerative diseases as an important DAMP [79]. Also, an animal study using anti-HMGB1 antibody revealed that neutralization of HMGB1 attenuates weight gain and liver inflammation, but not adipose tissue inflammation, under HFD feeding [14].

Another study, however, showed that TLR9 deficiency promoted insulin resistance in response to a HFD, suggesting anti-inflammatory roles of TLR9 in macrophage activation [13]. Several differences in the study design, including the diet and duration of feeding, might account for these discrepancies (Table 2). Further studies are required to elucidate the role of cfDNA-TLR9 signaling in the pathogenesis of metabolic disorders.

**Table 2:** Experiment models for exploring the roles of TLR9 in cardiometabolic disorders

Mice strain	Model (feeding)	Duration of feeding	Agonist	Antagonist	Role	Ref
Obesity						
*Tlr9*^*-/-*^ (B6.129P2^-Tlr9tmAki^)	HFD, 60 kcal% fat	12 weeks	-	-	promotive	[10]
C57BL/6		12 weeks	-	iODN2088 for 12 weeks		
*Tlr9*^*+/+*^ bone marrow in *Tlr9*^*-/-*^ (B6.129P2^-Tlr9tmAki^)		12 weeks	-	-		
*Tlr9*^*-/-*^ (C57BL/6J^-Tlr9M7Btlr^)	HFD, 60 kcal% fat	15 weeks	-	-	promotive	[11]
C57BL/6		10 weeks	-	IRS954 for 3 weeks		
C57BL/6J	Normal chow diet	8 days	CpG-ODN2395 for 8 days	-		
C57BL/6J	HFD, 40 kcal% fat	12 weeks	-	-	-	[12]
*ob/ob*	Standard chow diet	12 weeks	-	-		
*Tlr9*^*-/-*^ (B6.129P2^-Tlr9tmAki^)	HFD, 60 kcal% fat	8-10 weeks	-	-	protective	[13]
Atherosclerosis						
*Tlr9*^*-/-*^*Apoe*^*-/-*^ (C57BL/6 background)	WTD, 21% fat, 0.2% cholesterol +Ang II-infusion for 4 weeks	12 weeks	-	-	promotive	[19]
*Tlr9*^*-/-*^*Apoe*^*-/-*^ (C57BL/6 background)		12 weeks	-	iODN2088 for 4 weeks		
*Tlr9*^*+/+*^ bone marrow in *Tlr9*^*-/-*^*Apoe*^*-/-*^ (C57BL/6 background)		12 weeks	-	-		
Apoe*3Leiden transgenic mice	WTD + cuff placement for 2 weeks	5 weeks	-	TLR7/9 dual antagonist for 2 weeks	promotive	[22]
*Apoe*^*-/-*^ (C57BL/6 background)	Cholesterol rich diet, 21% fat and 1.25% cholesterol	8 weeks	CpG-ODN1826 for 7 weeks	-	promotive	[26]
*Apoe*^*-/-*^	HFD, 21% fat and 0.15% cholesterol	6 weeks	CpG-ODN1585 for 5 weeks	-	promotive	[20]
*Tlr9*^*-/-*^*Apoe*^*-/-*^ (C57BL/6 background)	HFD, 21% fat and 0.15% cholesterol	12 weeks	-	-	protective	[27]
*Apoe*^*-/-*^ (C57BL/6 background)		8 weeks	CpG-ODN1668 for 8 weeks	-		

## Role of TLR9 in cardiovascular diseases

Chronic inflammation in the vasculature, initiated by endothelial dysfunction under risk factors such as hypertension, diabetes mellitus, and dyslipidemia, causes atherosclerosis [80]. Controlling these risk factors reduces cardiovascular events; however, considerable residual risk remains and is a clinical issue. This also indicates that the mechanisms that cause vascular inflammation and atherosclerosis are not fully understood.

Accumulating evidence indicates that the innate immune system plays a role in the development of vascular inflammation despite it being multifactorial in etiology [81]. Many types of PRRs are expressed in multiple cell types present in arterial lesions, including endothelial cells and infiltrated monocytes, macrophages, and dendritic cells. TLRs are essentially associated with the process of atherosclerosis [82]. Both in murine and human lipid-rich atherosclerotic lesions, macrophages show TLR4 expression preferentially, which is upregulated by oxidized low-density lipoprotein (oxLDL) [83]. Other studies showed that TLR2 expression is enhanced in patients with diabetes, and TLR2/TLR4 stimuli promote inflammation in obese patients with atherosclerosis [84, 85].

**Fig. 4:**
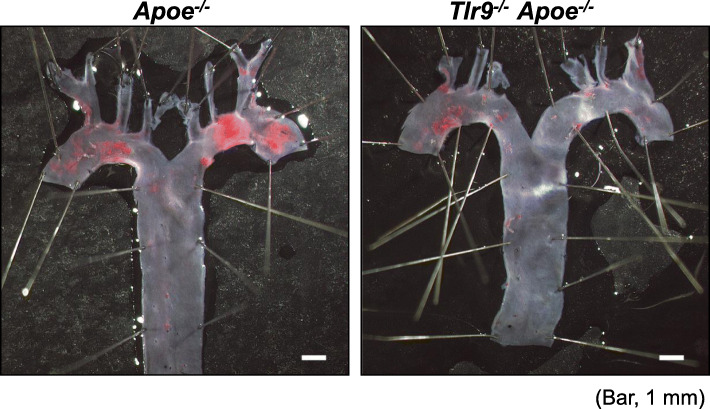
Genetic deletion of TLR9 attenuated the development of atherosclerosis. Representative figures of Sudan IV staining of the aortic arch of Ang II-infused *Apoe*^*−/−*^ or *Tlr9*^*−/−*^*Apoe*^*−/−*^ mice. Genetic deletion of TLR9 attenuated the development of atherosclerosis. Bar, 1 mm

In addition to TLR2 and TLR4, recent studies have suggested the contribution of TLR9 to the development of vascular inflammation and atherogenesis. In vitro studies demonstrated that the activation of TLR9 accelerates the shift from macrophages into foam cells via the NF-κB and IRF7 pathways [23, 24]. Also, we found that a TLR9 agonist, ODN1826, markedly promoted the pro-inflammatory activation of apolipoprotein E-deficient (*Apoe*^*−/−*^) macrophages, partially through p38 MAPK signaling [19]. Another study demonstrated the activation of plasmacytoid dendritic cells (pDCs) through the TLR9 pathway, leading to the development of vascular lesions [25]. Previous studies have shown degeneration of vascular cells including endothelial cells and macrophages in atherosclerotic lesions [86–88], suggesting the release of cellular debris that contains various endogenous ligands for TLRs [89]. Therefore, we hypothesized that TLR9 plays a role in the development of atherosclerosis through the recognition of DNA fragments released by vascular damage. To address this hypothesis, we employed three different mouse models. Genetic deletion of TLR9 in subcutaneous angiotensin II (Ang II)-infused *Apoe*^*−/−*^ mice on a Western-type diet (WTD) reduced the development of atherosclerotic lesions (Fig. 4). Pharmacological blockade of TLR9 using iODN2088, one of the inhibitory oligodeoxynucleotides specific to TLR9, attenuated atherogenesis in Ang II-infused *Apoe*^*−/−*^ mice compared with control oligodeoxynucleotide. Genetic deletion and pharmacological inhibition of TLR9 also decreased macrophage and lipid accumulation and the expression of inflammatory molecules at both the RNA and protein levels in this mouse model [19], while restoration of TLR9 in the bone marrow in *Tlr9*^*−/−*^*Apoe*^*−/−*^ mice accelerated atherogenesis in the aortic arch. These findings indicate proatherogenic roles of TLR9 [19]. Furthermore, Ma et al. showed that the inactivation of TLR9 by employing IRS869, another type of inhibitory oligodeoxynucleotide for TLR9, reduced plaque burden and shifted macrophage polarization to the anti-inflammatory M2 population [90]. Krogmann et al. also showed that intravenous administration of ODN1826 to *Apoe*^*−/−*^ mice impaired reendothelialization of an acute vascular injury and increased subsequent atherosclerotic plaque development [26]. Similarly, we have reported the contribution of TLR9 activation to neointima formation after mechanical vascular injury, which was blocked by the administration of an anti-HMGB1 antibody [21]. In addition, we also demonstrated that TLR9 activation impaired blood flow recovery in an ischemic hind limb model by the promotion of TNF-α expression [91]. All of these studies suggested TLR9 activation promotes inflammation and accelerates atherosclerotic and/or vascular diseases.

Recent clinical studies also indicated the contribution of the cfDNA-TLR9 axis in the development of atherosclerosis in humans. Borissoff et al. demonstrated that patients with severe coronary artery disease diagnosed by coronary computed tomographic angiography have elevated plasma dsDNA and nucleosome levels [18]. We also measured the level of cfDNA in the plasma collected from the target vessel of patients with acute myocardial infarction and examined the correlation between the concentration and plaque morphology of the target lesion assessed by optical coherence tomography. The plasma level of cfDNA in the target vessel was positively correlated with lipid deposition, macrophage content, and ruptured plaque cavity length/area in the target lesion, all of which are associated with plaque inflammation [19]. Several studies have shown the expression of TLR9 in vascular lesions, although its expression may be lower than that of other TLRs such as TLR4 and TLR2 [82]. However, these studies suggested that the cfDNA-TLR9 axis participates in the pathogenesis of vascular inflammation and atherogenesis.

On the other hand, several groups have reported incongruous findings which suggested protective roles of TLR9 in atherosclerosis. In vitro stimulation of TLR9 triggered IL-10 production in B cells in humans, which in turn inhibited CD4^+^CD25^+^ T cell proliferation [92, 93]. Also, Koulis et al. demonstrated antiatherogenic roles of TLR9 using a genetic deletion model of TLR9 in HFD-fed *Apoe*^*−/−*^ mice [27]. In that study, TLR9-deficient *Apoe*^*−/−*^ mice showed increased macrophage, DC, and CD4^+^ T cell in the plaque. Also, the administration of CpG-ODN1668, a TLR9 agonist, attenuated atherosclerotic lesion development in *Apoe*^*−/−*^ mice under HFD feeding. Of note, the study of Koulis et al. showed that TLR9 deletion increased blood lipid levels by an undetermined mechanism. This might have affected the results and suggested that TLR9 is associated with lipid metabolism in addition to the innate immune system. Thus, both pro- and anti-atherosclerotic roles of TLR9 have been described. Interestingly, a previous study mentioned conflicting roles of TLR9 activation due to the concentration of its ligand [94]. Therefore, the difference in the study design such as mouse model, mouse strain, food, duration of treatment, and types of agonist or antagonist might cause the difference in the levels of ligands, which results in these discrepancies observed in previous studies (Table 2). Further experiments are needed to determine the effect of TLR9 in atherosclerotic diseases.

Accumulating evidence suggests that cardiac inflammation contributes to promoting heart failure (HF). In HF patients, levels of circulating cytokines including TNF-α, interleukin (IL)-1β, and IL-6 are elevated, which is associated with the severity and outcome of these patients [95]. In most cases, microbial infection is not involved in the development of HF, indicating that there is a state of sterile inflammation. However, the complex mechanisms underlying cardiac inflammation are unclear [96]. In HF, multiple endogenous DAMPs such as HMGB1, HSP, and mtDNA are released and recognized by TLRs, stimulating NF-κB-dependent inflammatory responses [97]. Recently, a study demonstrated that intracellular mtDNA escaping degradation induces cardiac inflammation signaling through TLR9 in an animal model of pressure overload-induced HF [28]. Endogenous mtDNA in the extracellular space activates NF-κB signaling through TLR9 in cardiomyocytes, resulting in its detrimental effects [29]. In addition, inhibition of TLR9 attenuated the development of pressure overload-induced HF [98].

Thus, recently, the role of TLR9 is expanding to the cardiovascular field as well as the innate immune field. Investigating the role of the cfDNA-TLR9 axis would increase the understanding of the pathogenesis and generation of new therapeutic approaches for these diseases.

## TLR9 as a therapeutic target for inflammatory diseases

Because recent advances of immunology indicated the role of TLRs in various inflammatory diseases, TLRs are receiving increased attention as the therapeutic target. Previously, two phase III clinical trials using TAK-242, a small molecule which targets TLR4, were carried out [99]. The first trial (NCT00143611) resulted in not enough satisfaction because of failure to effectively decrease serum cytokine levels (IL-6, IL-8, and TNF-α) compared to controls, in spite of its well-tolerance [100]. Another trial (NCT00633477) ended because of a business decision. Since then, TAK-242 has not been developed clinically.

In contrast, drugs targeting endosomal TLR attract much attention. A group of antimalarial drugs, such as chloroquine (CQ), hydroxychloroquine sulfate (HCQ), and quinacrine, have been used to treat autoimmune diseases in clinical practice [101]. These drugs are weak bases that accumulate in the acidic intracellular compartment such as endosomes and lysosomes, and modulate the pH in these vesicles, leading to the suppression of autoantigen presentation and inhibition of endosomal TLR signaling (TLR7, 8, and 9) [102]. In addition to the effects for autoimmune diseases, pre-clinical studies suggested beneficial effects of these drugs on cardiovascular diseases. For example, pretreatment with CQ improved cerebral ischemia symptoms in a transient global cerebral ischemia rat model animal [103], and long-term treatment with HCQ attenuated hypertension and endothelial dysfunction in a lupus animal model as well [104]. Oligonucleotides with specific sequences also function as antagonists of endosomal TLRs because endosomal TLRs recognize nucleic acid structures. These oligonucleotides can block the TLR signal transduction by inhibiting the binding of TLRs to their ligands. Because of this background, variety types of these oligonucleotides have been developed for the treatment of autoimmune diseases including SLE and plaque psoriasis in both basic and clinical researches [105, 106].

Thus, controlling TLR9 signaling might have a potential to inhibit cardiometabolic diseases. In fact, previous studies have suggested that several types of oligonucleotides for TLR9 have inhibitory effects on the development of cardiometabolic diseases in animal models (Table 2). In addition, other studies have suggested that antibodies targeting HMGB1 or CD4 attenuate TLR9-mediated atherogenesis [27, 107]. Targeting immune responses mediated by TLR9 has a potential as a therapeutic strategy to control unwanted, disease-associated inflammation; however, further studies are needed to develop therapeutic strategies targeting immune systems clinically.

## Conclusion

**Fig. 5:**
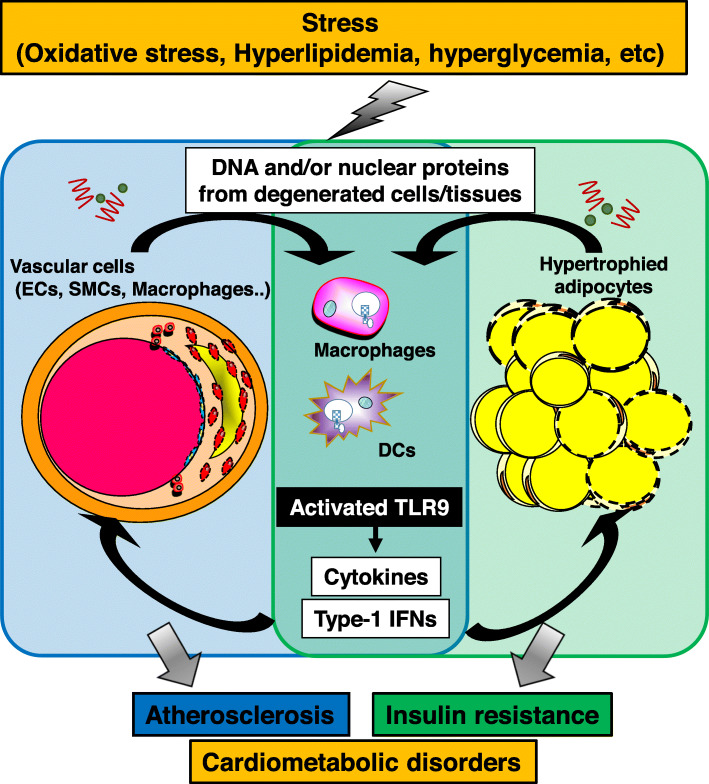
Role of TLR9 in the development of cardiometabolic disorders. DNA fragments and/or nuclear proteins released from damaged cells/tissues activate immune cells such as macrophages and DCs through TLR9, leading to the development of inflammation in these tissues, which is central in the pathogenesis of cardiometabolic disorders

Chronic low-grade inflammation plays a central role in the pathophysiology of cardiometabolic disorders, in which various cellular and molecular mechanisms participate. Recent studies have suggested that activation of innate immune systems by DAMPs contributes to the development of chronic inflammation [89]. The present review focused on the role of TLR9, which was originally known as a sensor for exogenous DNA fragments, in pro-inflammatory activation of immune cells and in the pathogenesis of cardiometabolic disorders. Innate immune systems are essential for survival. Originally, inflammation induced by sensing of DNA fragments has a protective role, although emerging evidence demonstrated that this immune system also has harmful effects. The prevalence and incidence of metabolic disorders associated with aging, obesity, and nutritional excess have dramatically increased worldwide in recent decades. This change may induce a shift in what are usually favorable for physiological processes to pathological events. Recent studies including our own demonstrated that the cfDNA-TLR9 pathway plays a pivotal role in the pathogenesis of adipose tissue inflammation and vascular inflammation via pro-inflammatory activation of macrophages, leading to the development of cardiometabolic disorders including obesity-related insulin resistance and atherosclerosis (Fig. 5). Regardless of accumulating research these days, there is still limited knowledge about the cfDNA-TLR9 pathway in cardiometabolic fields. For example, the origin of elevated DNA fragments in obese and atherosclerotic conditions is still not clear. The release of nucleic acids as cellular debris from degraded cells/tissues in metabolic organs together with other TLR agonists is considered to be one of them. However, recent studies have shown a link between the gut microbiota and metabolic disorders [17, 108, 109]. These studies suggested the translocation of bacterial components (such as DNA) and inflammatory factors (including lipopolysaccharide) in the host circulation under certain circumstances, accompanied by intestinal epithelial dysfunction caused by obesity or other metabolic disorders.

In summary, the cfDNA-TLR9 pathway contributes to the pathogenesis of cardiometabolic disorders. This pathway might be a potential therapeutic target and a possible biomarker for this health threat. However, further studies are required to define the possible clinical application of this pathway.

## Data Availability

The datasets during and/or analyzed during the current study are available from the corresponding author on reasonable request.

## Abbreviations

Ang II: Angiotensin II
Apoe^−/−^: Apolipoprotein E-deficient
cfDNA: Cell-free DNA
CQ: Chloroquine
DAMPs: Damage-associated molecular patterns
dsDNA: Double-stranded DNA
ER: Endoplasmic reticulum
HCQ: Hydroxychloroquine
HF: Heart failure
HFD: High-fat diet
HMGB1: High mobility group box protein-1
HSP: Heat shock protein
IFN: Interferon
IL: Interleukin
IRF: Interferon regulatory factor
MAPK: Mitogen-activated protein kinase
MCP-1: Monocyte chemoattractant protein-1
MDA5: Melanoma differentiation-associated gene 5
mtDNA: Mitochondrial DNA
MyD88: Myeloid differentiation primary response
NASH: Non-alcoholic steatohepatitis
oxLDL: Oxidized low-density lipoprotein
PAMPs: Pathogen-associated molecular patterns
pDCs: Plasmacytoid dendritic cells
PRR: Pattern recognition receptor
RIG-I: Retinoic acid-inducible gene I
SLE: Systemic lupus erythematosus
ssDNA: Single-stranded DNA
STING: Stimulator of interferon genes
TLR: Toll-like receptor
TNF-α: Tumor necrosis factor-α
WTD: Western-type diet
